# A dynamic Bayesian network approach to modeling engagement and walking behavior: insights from a yearlong micro-randomized trial (*Heartsteps II*)

**DOI:** 10.1080/21642850.2025.2552479

**Published:** 2025-09-18

**Authors:** Steven A. De La Torre, Mohamed El Mistiri, Karine Tung, Eric Hekler, Predrag Klasnja, Misha Pavel, Daniel E. Rivera, Donna Spruijt-Metz, Benjamin Marlin

**Affiliations:** a The Herbert Wertheim School of Public Health and Human Longevity Science, University of California, San Diego, CA, United States; b Control Systems Engineering Laboratory, School for Engineering of Matter, Transport, Energy, Arizona State University, Tempe, AZ, USA; c Manning College of Information and Computer Sciences, University of Massachusetts Amherst, Amherst, MA, USA; d Herbert Wertheim School of Public Health and Human Longevity Science, University of California, San Diego, CA, USA; e Design Laboratory, University of California, San Diego, CA, USA; f Center for Wireless and Population Health Systems, University of California, San Diego, CA, USA; g School of Information, University of Michigan, Ann Arbor, MI, USA; h Khoury College of Computer Sciences, Northeastern University, Boston, MA, USA; i Bouve College of Health Sciences, Northeastern University, Boston, MA, USA; j Dornsife Center for Economic and Social Research, Department of Psychology, University of Southern California, Los Angeles, CA, United States

**Keywords:** Dynamic Bayesian networks, digital behavior change interventions, mobile health (mhealth), physical activity, user engagement, longitudinal data analysis, dynamic theories of behavior change

## Abstract

Introduction: Mobile health (mHealth) technologies such as wearable activity trackers (e.g. Fitbit) and digital applications (apps), can support behavior change in real-world contexts. Since effectiveness is dependent, in part, on participants’ engagement with the digital technology (e.g. app page views) and the intervention components (e.g. anti-sedentary messages), there is a need for modeling approaches that support the investigation of engagement in digital interventions and the refinement of dynamic theories of behavior change. Methods: Dynamic Bayesian Networks (DBN) were used to model the idiographic (individual) dynamic relationships between a participant’s daily app engagement (page views), walking behavior, and intervention messages, accounting for context (e.g. temperature), and psychological variables (e.g. perceived restedness and perceived busyness). Additionally, we explored differences in the resulting DBN models between participants of Hispanic/Latino and non-Hispanic/Latino White backgrounds. Results: Data from 10 participants in the HeartSteps II study (n = 5 Hispanic/Latinos and n = 5 non-Hispanic/Latino Whites) was used. Across participants (100%, n = 10), there was a strong positive effect of the number of messages/prompts received on their daily app page views with a predicted increase range of 12.84 (12.19–13.57) to 25.84 (24.28–27.59) app page views per day per message received. Among the majority of Hispanic/Latino participants (n = 4/5, 80%), there was a strong positive relationship between daily app page views and walking behavior with predictions ranging from a mean of 6.70 (6.37–7.05) to 10.93 (10.14–11.78) steps per minute of Fitbit wear time per app page view. Both groups showed idiographic differences in the effects of temperature and perceived busyness on walking behavior. Conclusion: The results demonstrate the benefits of DBNs to model the daily-level idiographic behavioral dynamics of engagement in digital intervention studies. This approach can be leveraged to support the refinement of dynamic theories of behavior change and improving personalized mHealth intervention strategies.

## Introduction

Barriers to physical activity such as low motivation or perceived lack of time may inhibit individuals from engaging in regular exercise (Trost et al., 2002). Inactive individuals who experience these phenomena may not be aware of how or when they might be able to integrate physical activity, such as walking, into their day. Mobile health (mHealth) technologies such as wearable activity trackers (Fitbit, Garmin, Apple Watch, etc.), often in conjunction with digital applications (apps) may be particularly useful for encouraging behavior change and aiding individuals in identifying opportunities for exercise in their given context. These technologies can deliver ‘mini–interventions’ in the form of prompts such as anti-sedentary messages, used to remind individuals to perform a behavior or task, motivational messages, or, providing opportunities for self-monitoring and reflection (Michie et al., 2011). Additionally, follow-up prompts can be delivered after an individual has established a routine with the aim of reminding them to maintain the behavior change or achieve a specific health goal. Ultimately, as the person becomes better at performing the established behavior over time, reminders and prompts can be reduced (Michie et al., 2011).

Despite the potential upside of this message-based approach, the literature provides mixed evidence of their effectiveness to impact sustained behavior change (Stawarz et al., 2015; Tobias, 2009). One alternative to the more ‘straight-forward’ forms of prompts or notifications mentioned previously is to incorporate adaptive, context-aware reminders, sometimes referred to as Just-in-Time (Moller et al., 2017; Nahum-Shani et al., 2018). Just-in-Time reminders can be leveraged to support both habit formation (prompting individuals to engage in a target behavior in an optimal context) and habit breaking (prompting individuals to refrain from an unwanted behavior) (Pinder et al., 2018) providing a flexible method for behavior change. However, there is need to better understand the impact of varying contextual conditions on engagement with these intervention prompts and their impact on behavior.

Second, participant burden is a major concern in mHealth studies. Some of the most common forms of burden in these studies is the demand to engage in self-report, such as answering Ecological Momentary Assessment (EMA) (Stone and Shiffman, 1994) surveys, or the demand of responding to intervention prompts or messages that are delivered (Nahum-Shani et al., 2022). The concern is that the high demand placed on individuals in mHealth studies through these mechanisms can become too burdensome and lead to eventual habituation. Habituation in this context is commonly described as a decrease in behavioral response resulting from over stimulation (Rankin et al., 2009; VandenBos, 2007). Therefore, if excessive or non-relevant message delivery can potentially have a negative impact on engagement, which may lead to burnout or dropout, then it is paramount to understand the impact of potential burden (e.g. total number of prompts and messages sent) on Digital Behavior Change Intervention (DBCI) engagement and walking behavior, as well as the contextual conditions that afford or undermine engagement and ultimately, behavior. This is of importance because, despite their demonstrated efficacy (McLaughlin et al., 2021; Stockwell et al., 2019), the effectiveness of DBCIs to impact behavior is reliant, in part, upon user engagement with the devices and intervention prompts designed for behavior change (Donkin et al., 2011; Smith and Liu, 2020).

In addition to prompt or message delivery, mHealth technologies and sensors can be leveraged to detect contextual conditions (e.g. location, temperature, psychological state) to identify when an individual participant’s conditions are suitable for effective behavior change. This allows for a critical opportunity to collect longitudinal, individual-level data on these conditions that can be used to build and test dynamic theories and models of behavior change at the individual (idiographic) and group (nomothetic) levels.

In this study, we selected contextual and psychological variables that were both theoretically relevant and able to be captured or aggregated to the daily level to support our modeling efforts. Specifically, we included data on participants’ self-reported perceived busyness and perceived restedness as two indicators of their daily context. These constructs align with existing behavior change theories including the Temporal Self-Regulation theory, which posits that self-regulatory capacity and daily self-regulatory context (e.g. stress, time scarcity) impacts the relationship between intention and behavior (Hall and Fong, 2015). In this sense, the perceived busyness variable represents the daily perceptions of time pressure as well as cognitive burden, and the perceived restedness variable reflects an individual’s feeling of energy and cognitive readiness to engage with both the digital intervention platform and physical activity behavior that day. These variable selections were also supported by previous work on idiographic modeling efforts using longitudinal data and similar intervention designs which highlight the impact of contextual factors on physical activity and DBCI engagement (De La Torre et al., 2024; El Mistiri et al., 2024; Phatak et al., 2018) as well as studies supporting the focus on daily-level analysis on walking behavior (Chevance et al., 2021a, 2021b).

We recognize that existing psychological and behavioral theories can provide a structure and framework to investigate behavioral and psychological phenomena, as well as to generate and test hypotheses of behavior change (Calder et al., 2023). However, these theories typically present unidirectional, static relationships between influences and behavior, and often fail to capture the complexity of an individual’s real-world behavior, in context, over time (Eronen and Bringmann, 2021; Guest and Martin, 2021; Oberauer and Lewandowsky, 2019; Perski et al., 2024). Furthermore, the methods used to test these models primarily focus on providing insights into ‘if’ there are relationships or associations between variables but tell us little about ‘how’ or ‘why’ these variables are related to each other through time mechanistically, or revealing their underlying processes.

The current understanding of DBCI engagement comes predominantly in the form of ‘snapshots’ of behavior, and does not capture its inherently dynamic nature, which evolves and responds to an individual’s ever-changing contextual conditions (Spruijt-Metz et al., 2015). Due to the rise in use of digital tools and sensors, we can capture repeated measurements of an individual’s behavior and context over time, also known as intensive longitudinal data (ILD). Given this increased ability to capture ILD, we have an exciting opportunity to leverage this data to test dynamic models of behavior change, to test and refine dynamic theories, and in our case, test and develop dynamic theories of DBCI engagement that apply to individuals and potentially groups.

## Methods

### *Heartsteps II* intervention overview

This study leveraged data from the NIH-funded study ‘Operationalizing Behavioral Theory for mHealth: Dynamics, Context, and Personalization’ (also known as ‘*HeartSteps II*’). *HeartSteps II* is a year-long Micro–Randomized Trial (MRT) that utilizes a wearable activity tracker to promote walking behavior and physical activity among overweight but otherwise healthy adults in Southern California. The full design of the *HeartSteps II* MRT is described in the protocol paper (Spruijt-Metz et al., 2022). Additional information about *HeartSteps II* relevant to this study, including study measures, are included in **Appendix A**. Ten participants were selected from the *HeartSteps II* study based on pre-specified data sufficiency criteria necessary to support our modeling efforts and estimation including: (1) having less than 40% missing data across key behavioral and psychological measures, and (2) sufficient within-person intraday variability in these measures. This study was conducted according to the guidelines of the Declaration of Helsinki, and approved by the Institutional Review Board of The University of Southern California (protocol code UP-18-00791, date of approval 5 December 2018). Informed consent was obtained from all subjects.

### Dynamic theory construction

**Figure 1.**
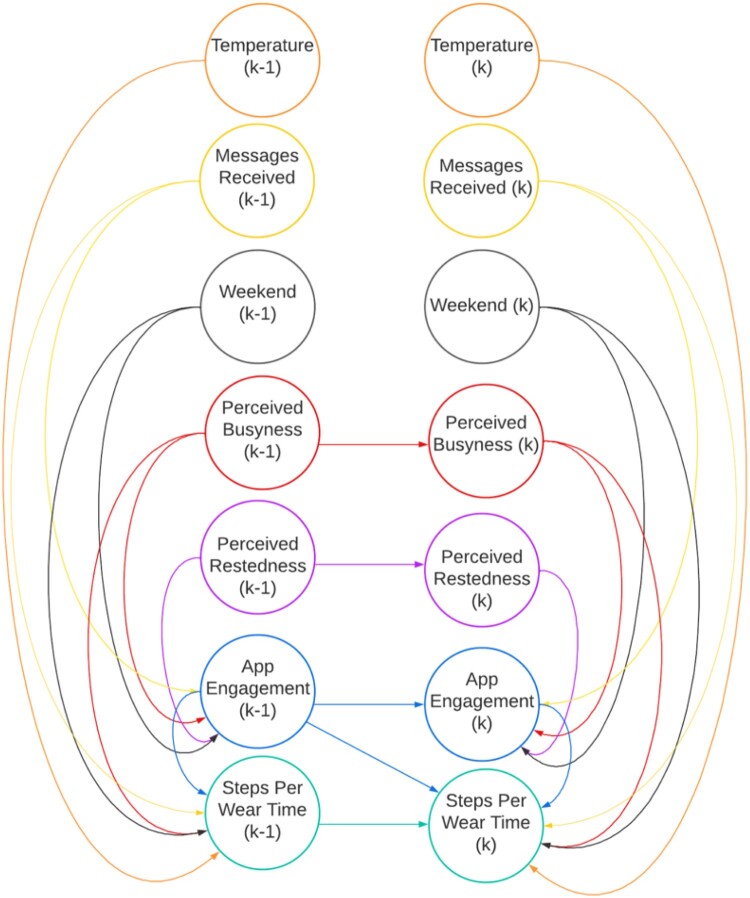
Dynamic Bayesian Network (DBN) model of daily-level, idiographic behavioral dynamics of intervention components, walking behavior, app engagement, and psychosocial and behavioral measures. **Long Description:** A Dynamic Bayesian Network (DBN) model illustrating time–lagged relationships between seven variables. Each variable is represented by a circle, with arrows showing directional dependencies. The variables include temperature, messages received, weekend status, perceived busyness, perceived restedness, app engagement, and steps per wear time minute at two-time points k-1 and k. Variables from the earlier time point influence their counterparts and other variables at the same time point and the future timepoint.

To address the research gaps outlined in this paper, we utilized the Theory Construction Methodology (TCM) (Borsboom et al., 2021) in which we aim to iteratively generate, test, and refine dynamic behavioral theories using idiographic modeling and ILD. Rather than testing a single a priori theory, we use the TCM approach to uncover context-sensitive mechanisms that impact DBCI engagement and physical activity at the daily level that have both theoretical and DBCI design implications for Just-in-Time interventions. The TCM describes five high-level steps utilized in this study including: (1) specifying the empirical phenomena of interest, which in our case is DBCI engagement, rooted in data collected in the *HeartSteps II* study, (2) formulating a prototheory (represented graphically in Figure 1), which specifies the mechanistic causal relationships among the observed variables using an abductive approach, (3) translating our prototheory into a formal, mathematical model (described below in the form of difference equations), (4) testing the mathematical model using Bayesian estimation approaches, and (5) evaluating the model’s ability to represent the observed phenomena, allowing us to investigate which dynamic hypotheses were relevant for which participant. Overall, TCM provided a high–level structure for our work to investigate daily-level idiographic behavioral dynamics presented in this manuscript.

### Analytic approach – Bayesian networks

Bayesian networks (BNs), which are a type of probabilistic graphical model, are represented by: (1) a set of variables and (2) specifying their conditional dependencies. This is typically depicted through a directed acyclic graph (DAG) (elaborated on below). Due to recent advances in computational algorithms, Bayesian analysis has now been applied to the modeling of complex behavioral and psychological modeling processes that can support real–world decision making (Kyrimi et al., 2021).

Underlying Bayesian networks and inference is Bayes theorem, which is a formula that enables researchers to calculate and update an initial belief in the probability of an unknown hypothesis when new evidence is observed (Bayes, 1763). The updated probability is referred to as the posterior probability, which is conditional on the evidence being observed (Neapolitan and Jiang, 2007). BNs formalize this idea by representing the variables (e.g. behavioral, psychological, contextual) as a DAG which combines graphical representation and parameterization. Using this method, the graphical representation (DAG) provides the structure of the BN, which is comprised of nodes that represent random variables (discrete or continuous) and directed arcs which represent direct probabilistic influence of one variable on another (Neapolitan and Jiang, 2007; Neuberg, 2003; Pearl, 2009; Pearl and Russell, 2003). For example, to represent the reception of an anti-sedentary message impacting the probability of the user breaking their sedentary state and engaging in a short walking bout would be represented as: anti-sedentary message → walking behavior. Both the BN structure and parameters can be specified using: (1) automated learning from data, if sufficiently available, (2) a ‘manual’ approach using domain knowledge (Julia Flores et al., 2011), or (3) a combination of both approaches, which was utilized in this study.

A BN consists of a set of parameterized conditional probability functions associated with each node that represent the conditional probability distribution of each node in the BN given its parents (or predecessors). The joint distribution of a set of variables is defined to be:

(1) $$\;\;P(X_{1}=x_{1},X_{2}=x_{2},\ldots ,X_{n}=x_{n})=\overset{n}{\underset{i=1}{\prod }}\;P(X_{i}=x_{i}|\mathrm{Pa}(X_{i})=\mathrm{Pa}(x_{i}))\;\;$$

where … $X_{1}$, $X_{2},\;\ldots \;X_{n}$ denotes a set of random variables (nodes), $x_{1}$, $x_{2},\;\ldots \;x_{n}$ denotes a set of values for each random variables, $\mathrm{Pa}(X_{i})\;$denotes the random variables corresponding to the parents of node i in the DAG, $P(X_{i}=x_{i}\mathrm{Pa}(X_{i})=\mathrm{Pa}(x_{i}))\;$denotes the conditional probability that node $X_{i}$ takes value $x_{i}$ given the value of its parents (Pearl and Russell, 2003). Since this temporal ordering specifies the direction of causality, we can extend this approach to the design of Dynamic Bayesian Networks (DBN).

### Dynamic Bayesian networks

While the BN approach can model the interdependencies between psychological and behavioral variables, BN-based analyses are typically time-invariant. BNs can essentially provide a snapshot of a system and the probabilistic relationships among a set of variables at a given timepoint. However, it does not provide information about how a variable, or variables, may be related to themselves, and other variables, at previous and future timepoints.

As an extension of BNs, DBN’s are utilized in this study to model the temporal dependencies between variables. In our case, understanding the underlying temporal dynamics of DBCI engagement is of primary importance. This was achieved by transforming the initial BN model into a DBN that incorporates effects over multiple time slices (e.g. time t-1, time t, time t + 1). The simplest form of a dynamical structure in this approach is for the probability distribution over the value of a random variable at time t + 1 to depend on the value of that variable at the previous time t (e.g. *X*(*t*) → *X*(*t* + 1)). Therefore, the initial BN was transformed into a DBN by taking the initial BN in DAG form and the initial probability distribution of these variables and adding temporal dependencies between variables at different time points (Neapolitan and Jiang, 2007) (Figure 1).

### Dynamic modeling approach

Behavior change processes often evolve over multiple time scales and may involve non-linear or threshold dynamics (e.g. learning, disengagement, relapse). However, many existing theories and models lack the temporal resolution to capture these fine-grained within–person processes. To establish a foundation for this line of work, we focused on modeling short–term, first–order lagged dependences using DBNs, which can support idiographic modeling and inform daily intervention adaptation. Although our current model assumes local stationarity, it establishes the foundation for future work to incorporate longer term dynamics using approaches like latent states, time–varying parameters, or structured time effects, among other approaches.

One of the advantages to using DBNs as a modeling approach is their ability to learn from data and incorporating a priori knowledge into the model. The process of building DBNs typically involves two parts. The first is determining the network structure (structure learning). Second, estimating the parameters that best describe the data given the network structure (parameter learning). Both processes to build the dynamic model in this study are described below.

### Structural learning

As mentioned above, in the DAG representation (Figure 1), nodes are represented as circles and correspond to random variables (e.g. prompts and messages received) or psychological constructs (e.g. intrinsic motivation) and the arrows between the nodes represent direct probabilistic dependence between time (t-1), time (t), and time (t + 1) which can reflect lead or lagged effects to variables at different timepoints. We tested several dynamic hypotheses in the DBN model that are specified graphically in Figure 1.

The dynamic hypotheses are also specified below:

1. Perceived Busyness [k] is influenced by Perceived Busyness [k-1].
2. Perceived Restedness [k] is influenced by Perceived Restedness [k-1]
3. Daily Application Page Views [k] is influenced by Daily Application Page Views [k-1], Perceived Busyness [k], Perceived Restedness [k], Weekend [k], Daily Total of Messages and Prompts Received [k], and Temperature [k]
4. Daily Fitbit Steps Per Wear Time [k] is influenced by Daily Fitbit Steps Per Wear Time [k–1], Daily Application Page Views [k], Daily Application Page Views [k-1], Perceived Busyness [k], Weekend [k], Daily Total of Messages and Prompts Received [k], and Temperature [k].

*k represents a time index (previously referred to as t in the introduction)

### Parameter learning

By integrating the DBN network structure described above and specified in Figure 1 and data from the *HeartSteps II* study, the goal of parameter learning is to infer the parameters of the hypothesized model. Due to missing data and to account for uncertainty in this study, approximate Bayesian inference methods were used. Approximate Bayesian inference handles unknown model parameters as random variables and approximates the posterior distribution over the model parameters given the observed data (Minka, 2001). The resulting posterior distribution captures the uncertainty in the unknown parameter values resulting from incomplete data. To support this approach, prior distributions of model parameters were specified, primarily informed by domain knowledge. Second, to compute the posterior distributions, Markov Chain Monte Carlo (MCMC) methods were used (Hoffman and Gelman, 2014; Neal, 2012). Using this approach, MCMC-based inference can be performed for the unknown model parameters and the missing data simultaneously under the assumption that the missing data are missing at random. Lastly, distributions for each variable were specified. In the tested models, all relationships were specified via linear conditional Gaussian distributions. The weight and bias model parameters were given normally distributed prior distributions and the standard deviation parameters were given exponential priors. For all models, 1000 sample iterations were utilized for model estimation and multiple imputation. All parameters and their corresponding hypothesized connections can be found in **Appendix B**.

### Difference form equations

Our initial assumption is that the dynamics of variables in this study can be represented by first-order linear difference equations. Based on our DAG, we specified the following forms for the conditional mean of each variable in the model:

(2) Perceived Busyness (B(t)) = $w_{B}B(t-1)\;+\;b_{B}$
(3) Perceived Restedness (R(t)) = $w_{R}R(t-1)+b_{R}$
(4) Fitbit Step Count Per Wear Time Minute (FS(t)) = $w_{\mathrm{FS}}\mathrm{FS}(t-1)+\;w_{\mathrm{FSPV}}\mathrm{PV}(t)+\;+\;w_{\mathrm{FSBU}}\mathrm{BU}(t)+$ $\;w_{\mathrm{FSW}}W(t)+\;w_{\mathrm{FSMS}}\mathrm{MS}(t)\;+\;w_{\mathrm{FST}}T(t)\;+\;b_{\mathrm{FS}}$
(5) App Page Views (PV(t)) = $w_{\mathrm{PV}}\mathrm{PV}(t-1)+\;w_{\mathrm{PVBU}}\mathrm{BU}(t)+w_{\mathrm{PVR}}R(t)+\;w_{\mathrm{PVW}}W(t)\;+$ $\;w_{\mathrm{PVMS}}\mathrm{MS}(t)\;+\;b_{\mathrm{PV}}$

B = Perceived Busyness

R = Perceived Restedness

PV = Daily App Page Views

FS = Fitbit Step Count Per Wear Time

MS = Messages and Prompts Sent

BU = Busyness

T = Temperature

W = Weekend/Weekday Indicator

### Missing data

Missing data among health, behavior, and psychological studies is prevalent. In fact, it is estimated that 95% of randomized control trial (RCT) studies reported some missing data (Bell et al., 2014; Karahalios et al., 2012). The most commonly used approach to handling missing data across prevention studies is complete cases analysis (Lang and Little, 2018). Data loss and handling missingness are particularly relevant for longitudinal DBCI studies. Two primary reasons for data loss in DBCI studies are mobile sensor failure or non-wear, and a lack of response to self-report EMA (Goldberg et al., 2021). If not handled properly, missing data can bias conclusions and negatively impact inference (Kang, 2013).

**Figure 2.**
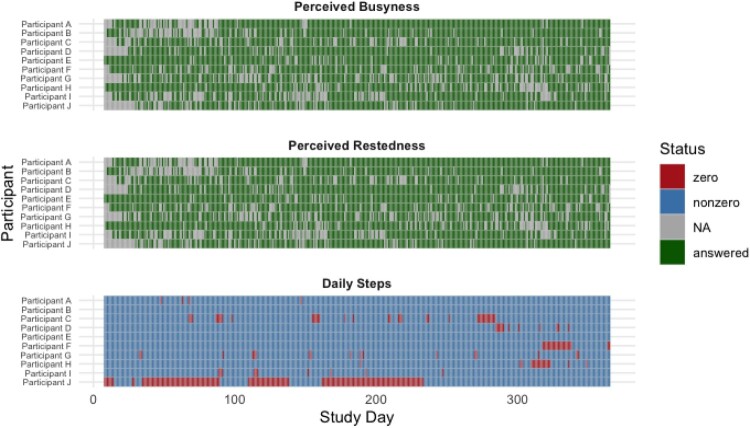
Heatmap of missing data across participants for perceived busyness, perceived restedness, and daily step count. **Long Description:** This figure provides a visual overview of data availability and completeness across three core variables collected daily from 10 participants in the *HeartSteps II* study. The top panel displays perceived busyness, the middle panel shows perceived restedness, and the bottom panel represents daily Fitbit step counts. Each row is a participant, and each column a study day. In the EMA panels (busyness and restedness), green tiles indicate days when participants completed the survey, and gray tiles indicate missing responses. In the step count panel, blue tiles show days with valid, nonzero Fitbit step data; red tiles indicate zero steps (often corresponding to non-wear or device syncing issues).

In this study, missing data were handled using a fully Bayesian joint modeling approach leveraging the capabilities of the *BayesLDM* toolbox (Tung et al., 2022) (described below). Rather than imputing values in a sperate preprocessing step, our approach marginalizes over missingness during parameter estimation, incorporating uncertainty from both data availability and model structure. This approach assumes data are Missing at Random (MAR), conditional on observed variables. Therefore, extended periods of missingness, which were limited amongst our data, result in wider posterior intervals, reflecting reduced confidence in model predictions. To further ensure model stability, we included only participants with less than 40% missingness across the variables used in these analyses and sufficient within–person variability to support dynamic estimation, as previously mentioned. Additionally, one of our primary variables of interests, Daily App Page Views, was fully observed. A visual heatmap of missing data amongst the participants is found in Figure 2.

### The BayesLDM toolbox

*The BayesLDM* toolbox utilizes a probabilistic modeling language that is specifically designed for modeling complex multivariate time series data and performing Bayesian inference on a specified model (Tung et al., 2022). These key features of *BayesLDM* can be leveraged to formally specify models using graphical representations (i.e. DAGs) and equations. Due to these features *BayesLDM* can handle model estimation, Bayesian inference, and Bayesian imputation in the same package. One of the key advantages in using the *BayesLDM* toolbox and a Bayesian imputation approach is the ability to account for posterior uncertainty when missing data is high (Gelman et al., 2013). Given the issues with missingness in DBCI studies, including *HeartSteps II*, leveraging the features of *BayesLDM* provides an excellent testbed for the application of this method to model ILD. Python version 3.7 was used for all analyses (Python Software Foundation, 2022). The full DBN program for one participant can be found in **Appendix C**.

## Results

### Descriptive statistics

Data from 10 participants in the *HeartSteps II* study (*n* = 5 Hispanic/Latinos and *n *= 5 non–Hispanic/Latino Whites) was used to estimate 10 individual DBN models. The average age of the participants in this sample was 46.30 (SD = 7.35) years, and their mean body mass index (BMI) was (31.54, SD = 5.15). Across participants selected for this study, the average steps per day at baseline was 6,239.16 steps (SD = 3,266.15) (Table 1). Results from a preliminary two–sample t-test, found that there was no significant differences between the two groups in their age (*p* = 0.09) or baseline steps (*p* = 0.06), however, there were significant differences in BMI (*p* = 0.03).

**Table 1.** Descriptive Statistics of the Study Sample (*n* = 10).

Variable	*n* (%)	Mean	SD	Median	IQR
**Overall**					
Age, years		46.30	7.35	47.00	7.75
Female	9 (90%)				
Hispanic	5 (50%)				
Body Mass Index (BMI)		31.54	5.15	30	6.8
Baseline physical activity, average steps per day at baseline		6239.16	3266.15	4938.07	3625.5
**Hispanic**					
Age, years		42.4	7.44	41	8
Female	5 (100%)				
Body Mass Index (BMI)		34.84	4.91	34.7	8.7
Baseline physical activity, average steps per day at baseline		4361.63	643.81	4457.43	844.43
**Non-Hispanic White**					
Age, years		50.2	5.31	50	4
Female	4 (80%)				
Body Mass Index (BMI)		28.24	2.91	27.50	0.2
Baseline physical activity, average steps per day at baseline		8116.69	3843.84	7932.71	3618

### Bayesian credible intervals

In Bayesian inference, a Bayesian Credible Interval (BCI) is an interval in which an unobserved parameter value falls within a particular probability (Hespanhol et al., 2019). BCIs are essentially analogous to confidence intervals in frequentist statistics, however, they differ in that BCIs treat their confidence bounds as fixed and the estimated parameter as a random variable, whereas frequentist confidence intervals used in statistics treat their bounds as random variables and the parameter as a fixed value (Hespanhol et al., 2019). These two intervals are also interpreted differently. In frequentist statistics, the interpretation of a confidence interval is that ‘there is 95% confidence that the true (unknown) estimate is within the lower and upper limits of the interval, based on hypothesized repeats of the experiment.’ Whereas the interpretation of the 95% BCI is ‘there is a 95% probability that the true (unknown) estimate is within the interval, given the evidence provided by the observed data’ (Hespanhol et al., 2019). In this study, Bayesian credible intervals were calculated via MCMC simulation described above.

### Model results: across participant findings

**Figure 3.**
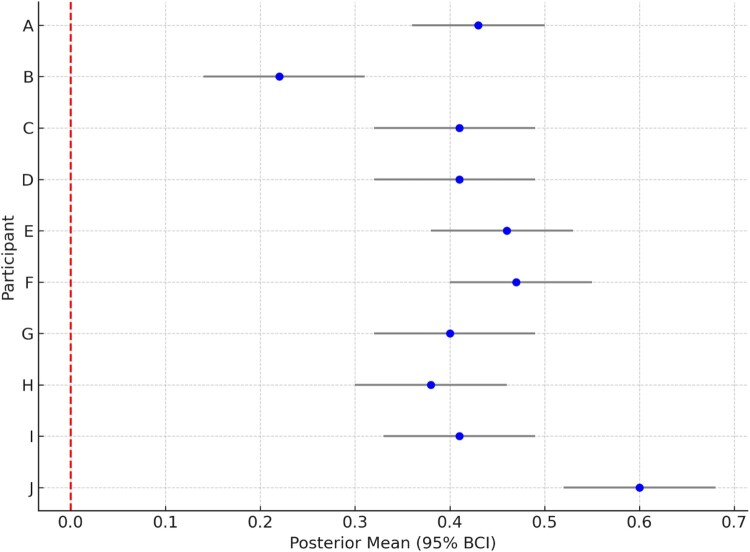
Posterior means and 95% Bayesian Credible Intervals (BCIs) for the effect of daily messages on app page views, by participant. Each point represents the estimated mean effect for one participant, and the horizontal bars indicate 95% BCIs. The red dashed line represents the null (zero effect). **Long Description:** This forest plot summarizes the estimated effect of daily messages received on app page views across 10 participants (labeled A to J). Each row shows a participant's posterior mean (blue dot) and 95% Bayesian Credible Interval (BCI) (black horizontal line). The vertical red dashed line at zero serves as a reference for no effect. Participants F and J show the largest positive estimated effects (∼0.4–0.6). The rest of the participants have intervals above zero, suggesting consistent positive associations between receiving messages and app engagement. This idiographic visualization highlights variation in effect magnitude across individuals.

***App page views:*** Across all participants (100%, n = 10), there was a strong positive effect of the *number of messages/prompts received by the participant [k]* on *daily application page views [k] (t_m)* with BCIs having almost all of their mass on positive values (not crossing or containing zero in the 95% BCI), ranging from a mean of 0.22 (SD = 0.05, 95% BCI: 0.14-0.31) to a mean of 0.60 (SD = 0.05, 95% BCI: 0.52-0.68) (Figure 3). This means that every message sent to these participants was associated with a predicted mean increase of 25.84 (24.28–27.59) and 12.84 (12.19–13.57) daily app page views respectively. Additionally, among most participants (9/10, 90%), the *weekend (t_ww)* had a positive impact on app page views with BCIs ranging from a mean of 0.1 (SD = 0.05, 95% BCI: 0.03-0.18) to 0.27 (SD = 0.05, 95% BCI: 0.19-0.35).

**Figure 4.**
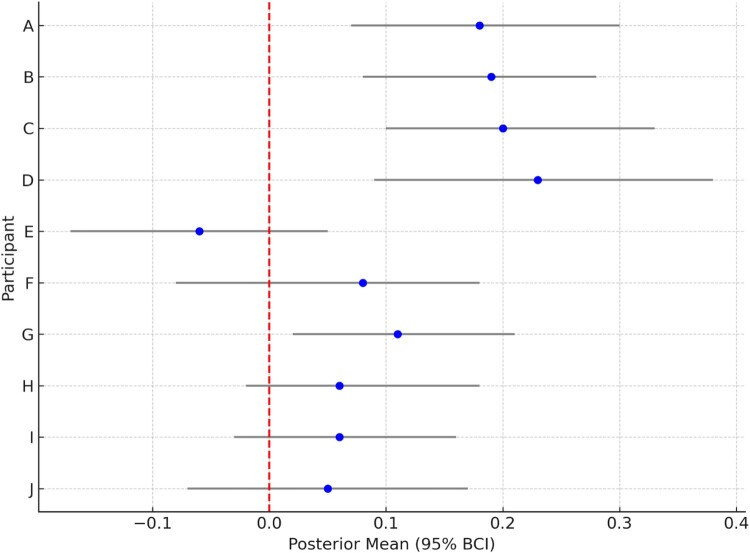
Posterior means and 95% Bayesian Credible Intervals (BCIs) for the mean effect of app page views on steps per wear time, by participant. **Long Description:** This figure summarizes idiographic Bayesian Credible Intervals (BCIs) of the relationship between daily app page views and Fitbit step counts across 10 participants. Each row corresponds to one participant, labeled A through J. Blue points denote the posterior mean of the estimated effect, and the black horizontal lines represent the 95% credible intervals (BCIs). The vertical dashed red line marks the null effect (zero).

**Walking behavior (Fitbit steps):** Among the majority of Hispanic/Latino participants (n = 4/5, 80%), there was a strong positive relationship between *daily app page views* and *walking behavior (t_fsaa)* with BCIs from individual models ranging from a mean of 0.18 (SD = 0.07, 95% BCI: 0.07-0.30) to 0.23 (SD = 0.09, 95% BCI: 0.09-0.38). In terms of steps, these values indicate that a 1-unit increase in app page views was associated with a predicted mean increase of 6.70 (6.37–7.05) to 10.93 (10.14–11.78) steps per minute when wearing the Fitbit (Figure 4). Among several participants (6/10, 90%) there was a direct, significant impact of *weekend (t_www)* on steps with BCIs from individual models spanning both positive values for some and negative values for other individuals ranging from a mean of −0.14 (SD = 0.05, 95% BCI: −0.22, −0.05) to 0.27 (SD = 0.08, 95% BCI: 0.15-0.4). This indicates that, according to these values, for one participant, the weekend was associated with significantly less mean steps per minute of Fitbit wear time and for another it was associated with a signficant increase. Overall, there was a small negative impact of *messages (t_msfs)* on steps across participants with BCIs ranging from a mean of −0.1 (SD = 0.05, 95% BCI: −0.2 –– −0.01) to −0.18 (SD = 0.06, 95% BCI −0.29 –– −0.09).

### Idiographic differences

Among both Hispanic/Latino and non-Hispanic/Latino White participants, there were significant idiographic differences in the effects of temperature on walking behavior (*t_t*). For example, one Hispanic/Latino participant had a significant positive relationship between temperature and daily Fitbit steps (mean = 0.12, SD = 0.05, 95% BCI: 0.04–0.21) and another had a significant negative relationship between temperature and steps (mean = −0.2, SD = 0.08, 95% BCI: −0.32 –– −0.05). In steps, that would equate to a predicted mean increase of 6.52 (6.29–6.78) steps per minute wearing the Fitbit for one participant and a decrease of 8.50 (7.82–9.35) for another, given a 1-unit increase in temperature.

A similar phenomenon was observed with the relationship between *perceived busyness and steps (t_baaa).* There were significant idiographic differences among non-Hispanic/Latino White participants with one participant having a negative relationship between the two (mean = −–0.17, SD = 0.06, 95% BCI: −0.25 –– −0.06) and another having a strong positive relationship (mean = 0.58, SD = 0.05, 95% BCI: 0.5–0.66), for example. This translates to a decrease in predicted mean steps per Fitbit wear time minute of 12.24 (11.59–13.12) for one participant and an increase of 16.96 (16.11–17.81) mean steps per Fitbit wear time minute, given a 1-unit increase in reported perceived busyness, highlighting this difference. Ultimately, these results would have not been identified without an idiographic modeling approaching and have significant implications for adaptive DBCIs.

### Exploratory differences by race/ethnicity

Although we did not explicitly formulate our model to test for ethnic or cultural moderation, we present disaggregated findings for Hispanic/Latino and non-Hispanic/Latino White participants to descriptively explore potential differences. These comparisons are exploratory and were not guided by pre-specified hypotheses.

**Figure 5.**
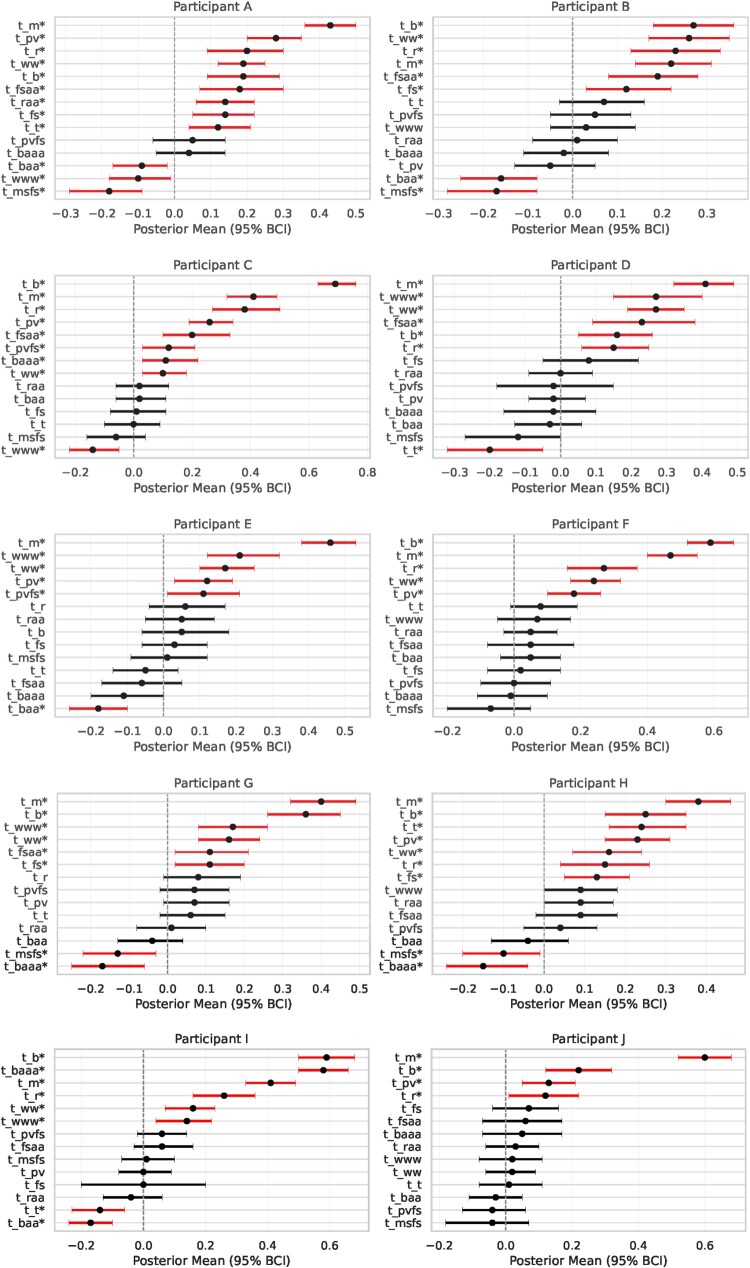
Results from idiographic dynamic models across participants. Posterior means and 95% Bayesian Credible Intervals (BCIs) are shown for each participant and predictor (labeled with abbreviated names). Parameters are sorted within each participant by effect size. **Long Description:** Multi-panel forest plots depict idiographic estimates of daily-level dynamic relationships between behavioral, contextual, and psychological variables across the 10 participants. Each subplot corresponds to a participant labeled A through J and includes horizontal lines showing the 95% Bayesian Credible Interval (BCI) for each t_ parameter. These parameters represent time-lagged relationships such as the influence of perceived busyness or app use on steps or app engagement that day or at the next time point. Posterior means are marked with dots, and intervals are color-coded: red intervals denote statistically credible effects (credible intervals that do not include 0), while black intervals denote non-significant estimates. Variables are ordered by effect size within each participant. The diversity of significant predictors across participants underscores the need for personalized modeling in adaptive behavioral health intervention studies.

For the majority of Hispanic/Latino participants (4/5, 80%) app page views (*t_fsaa*) had a positive impact on Fitbit steps per wear time on the same day compared to non-Hispanic/Latino Whites (1/5, 20%). However, interestingly, only among Hispanic/Latino participants (2/5, 40%), app page views from the previous day (*t_pvfs*) had an impact on steps the next day with a BCI mean of 0.12 (SD = 0.06, 95% BCI: 0.02 – 0.2). The impact of perceived busyness on application page views (*t_baa*) was more apparent for DBNs run on data from Hispanic/Latino participants vs. non-Hispanic/Latino Whites. Of the Hispanic/Latino participants, 60% (3/5), had BCIs that did not include zero containing negative values compared to only 20% (1/5) of non-Hispanic/Latino White participants, although the BCIs between the two groups were similar in magnitude and range. Conversely, perceived busyness on physical activity (*t_baaa*) had a more apparent impact for non-Hispanic/Latino Whites (3/5, 60%) compared to Hispanic/Latinos (1/5, 20%) and was mostly negative in direction. An idiographic summary of model estimates from the DBN model can found in Figure 5 and more information including a full list of MCMC parameters estimates across participants and additional posterior distribution plots can be found in **Appendices D-F**.

## Discussion

### General discussion

Across participants, the results from this study indicate that daily contextual factors such as being a weekend day, contextually dependent psychological factors such as perceived busyness, and daily intervention prompts or messages all had a significant impact on predicting daily app engagement and walking behavior. Additionally, similarities and differences in these relationships were found aggregated across people (overall), between groups (Hispanic/Latino vs. Non-Hispanic/Latino White), and between people (idiographic). These findings across multiple levels of aggregation provides supporting evidence for a ‘bottom-up approach’, starting with idiographic modeling, and then combining findings to make group–level inferences rather than focusing only on nomothetic analyses. This approach is similar to the increasing trend of moving toward precision medicine in healthcare, precision health as a larger concept, and personalized adaptive interventions in digital health (Denny and Collins, 2021; Hekler et al., 2020). Furthermore, an n-of-1 approach utilizing individual-level data and analytic techniques offers a pathway to gaining meaningful insights about key behavior change mechanisms that are relevant for an individual, and, potentially for larger groups, as investigated in this study, producing transportable and actionable knowledge in line with a ‘small data paradigm’, in contrast to big data approaches that center on group-level, data-centric findings (Hekler et al., 2019). By taking this approach, tailoring variables can be identified for individuals, and, for similar individuals with comparable behavioral patterns or non-time–varying characteristics (demographics, personality, etc.). Lastly, while the amount of intervention messages and prompts did not directly impact overall daily walking behavior among these participants, it had a positive impact on daily app engagement. Daily app engagement itself had a direct positive impact on walking behavior, particularly among Hispanic/Latinos. This provides a theoretical pathway for messages to impact walking behavior in other ways than they were designed to, as participants may have engaged in self-monitoring or reflection following the reception of a message, ultimately having a positive outcome on daily walking behavior.

### Potential racial/ethnic differences

While not central to our modeling objectives, we explored the dynamic relationships separately for Hispanic/Latino and non-Hispanic/Latino White participants due to the potential sociocultural differences in the impact of context-dependent psychological factors like how perceived busyness and perceived restedness may be experienced or acted upon. For example, prior work (described below) has suggested that cultural values around the use of time, family obligations, and rest can vary by race/ethnicity and may impact engagement. Therefore, our comparisons are strictly exploratory and descriptive and future work could explore these cultural moderators more explicitly.

The results from these modeling efforts found that the positive impact of daily app page views on daily walking behavior was more important for Hispanic/Latino participants compared to their non-Hispanic/Latino White counterparts. Perhaps this may reflect the importance of app engagement to overcome traditional barriers to engaging in PA for Hispanic/Latinos including lack of enjoyment from exercise, lack of knowledge on exercise performance, and experiences of discouragement (Bautista et al., 2011).

Additionally, the negative impact of perceived busyness on daily app engagement was more significant for Hispanic/Latinos vs. non-Hispanic/Latino Whites. When unpacking descriptive statistics to explore the potential for these differences, there were relatively equal distributions of responses to baseline questions on work autonomy between Hispanic/Latino and non-Hispanic/Latino Whites. However, Hispanic/Latino participants in the sample typically reported having a larger household size and having more children under the age of 12 compared to their non-Hispanic/Latino White counterparts. The existing literature also provides some insight. A qualitative study of middle-aged Hispanic women (n = 23), similar in demographics to those in this study, found that a major theme related to reasons for not engaging in PA was ‘family first, no time for myself’ and that these women perceived that PA was a ‘waste of time’ that didn’t fit into their busy schedules (Im et al., 2010). Other studies have also found that the one of the most frequently reported barriers to PA among Hispanic adults (n = 165/398, 41%) was ‘lack of time’ (Bautista et al., 2011). Perhaps, some of the differences in perceived busyness between the two groups is influenced by racial/ethnic differences in the experience of, perception of, and reporting of, perceived busyness.

### Idiographic differences

By utilizing a n-of-1, idiographic modeling approach, tailoring variables based on an individual’s respective context and patterns of behavior were identified. This finding is in line with other modeling work examining idiographic models of behavior change (De La Torre et al., 2024; Hall and Fong, 2015; Phatak et al., 2018). For example, the impact of temperature on daily app engagement and walking behavior had different effects both in magnitude and direction based on the individual participant. An interventionist might consider providing separate prompts and messages for folks who are more or less likely to be active given the current temperature. Also, given the findings of idiographic differences in the relationship between perceived busyness and daily walking behavior, identifying which participants have a negative relationship, and targeting them for supportive interventions while ignoring other participants whose perceived busyness doesn’t have a negative impact on their daily steps, could aid in reducing the risk of burden by delivering ineffective prompts causing experiences of annoyance or frustration, which may lead to disengagement.

### Advantages & limitations

In this study, a fully Bayesian approach leveraging the *BayesLDM* toolbox was used to address missing data among the participants. There are several advantages to this approach. First, by leveraging *BayesLDM,* model estimation and multiple imputation were performed simultaneously, reducing computational cost. Second, using a fully specified conditional model and specifying informed prior distributions injected domain knowledge about the hypothesized relationships, providing a theoretical foundation for the model. Lastly, this approach handled uncertainty regarding the properties of the missing data by estimating the posterior distributions over model parameters by marginalizing over missing data, which contrasts other commonly used methods such as interpolation (feed-forward algorithms), single (mean) imputation, deletion, complete cases analysis, among others. Ultimately, the results of this study provides evidence for the efficacy of using this approach to both efficiently handle data loss through Bayesian methods and execute the estimation of idiographic DBNs. Another key advantage of this study was the inclusion of a diverse sample including Hispanic/Latinos who are underreported and underrepresented in science and in mHealth studies, which was of paramount importance to the authors.

While there were several advantages to our approach, there were also limitations. The data used in this study was derived primarily from participants with sufficient information to support the dynamic modeling objectives. Therefore, the participants who were modeled in this study might have been more engaged than others who had higher rates of data loss due to poor EMA adherence, poor Fitbit wear time adherence, or study dropout. Second, the reliance upon the use of 5-item Likert scale EMA measures compared to more nuanced measures limited the investigation of modeling the daily-level dynamics of psychological constructs and objectively measured app engagement and walking behavior. To support these types of modeling efforts, there is a need in behavioral and psychological sciences to explore, establish, and utilize more sophisticated measurement approaches of psychological constructs to better support dynamic modeling.

We also recognize that our current model captures short-term behavioral dynamics under an assumption of local stationarity. This means that the modeled relationships are assumed to be stable over time within individuals. While this allows us to estimate interpretable lag-one effects, we acknowledge that behavioral change and adaption processes, such as learning, habit formation, disengagement, or relapse, likely involves utilizing longer term and nonstationary processes. Due to these factors, we were also not able to detect potential ceiling effects, particularly among those with relatively higher baseline steps, which may limit their potential for additional improvement to walking behavior. Future work can investigate how baseline behavior potentially moderates engagement with these types of interventions and whether cumulative effects unfold differently based on these characteristics.

Another limitation of our modeling approach is the assumption of MAR in the Bayesian framework. While we offer that this assumption is reasonable in the context of app engagement in this study, we cannot fully rule out non-random missingness due to disengagement or technical issues. For longer periods of missing data, the model reflects increased uncertainty in posterior estimates, but inference may still be biased if the MAR assumption doesn’t hold. Future work could explore extensions to handling missing data using Missing Not at Random (MNAR) to improve robustness.

### Future directions

As the field moves toward intervening on individuals, there is a strong need for idiographic modeling tools that can support causal inference and leverage the unique properties of ILD via mobile sensors. The results of this identifying daily-level contextual dynamics across different levels of aggregation can set the foundation for future researchers test and refine dynamic models to gain insights into underlying behavioral dynamics, in service of the development and refinement of dynamic theory, in a mathematically rigorous manner. The resulting updated dynamic behavioral theories in this line of work can help to explain behavior through time and in context. Having a better understanding of the underlying dynamics of a participant’s contextual environment and psychological state allows us to identify ‘windows of opportunity’ when intervention reminders and prompts are most effective. In doing so, investigators can minimize participant burden, maximize the effectiveness of prompts and messages, know which intervention components work best for which participants, and understand how these underlying processes unfold over time. This understanding is paramount to reduce the potential risk for ‘habituation’ to these types of intervention components such as message reminders (anti-sedentary messages, motivational messages, adherence messages, reminders, etc.) (Rankin et al., 2009). Given the nature of habituation, participants in DBCIs may be less inclined to respond to repeated messages or reminders, particularly those that don’t match their context, potentially leading to disengagement from the study, and perhaps without any potential for reengagement. Lastly, there is need to further develop methods that allow researchers to investigate the range of engagement experiences (e.g. disengagement, reengagement) when data on casual mechanisms may not available (e.g. no data on psychological or contextual variables). One approach is to integrate objectively measured time series data on app engagement with qualitative data from participants that investigates reasons for disengagement, if the participant is not completely lost to follow up. This integrated approach facilitates the contextualization of shifts between states of engagement (e.g. disengaged to reengaged) that are not possible through traditional measurement approaches. Furthermore, we recognize that extensions of our model can be made to explore processes such as disengagement, relapse, learning, and habit formation, by including time-varying parameters, polynomial or non-linear time terms to capture growth and decay dynamics, as well as latent state transitions through Hidden Markov Models (Du Roy et al., 2020) or segmented models to observe how associations evolve across different phases of an intervention, such as a Model-on-Demand (MoD) approach (Banerjee et al., 2025).

## Conclusions

In conclusion, this study presents an exploration of daily-level idiographic behavioral dynamics using fully Bayesian methods to test and refine dynamic theories of behavior change related to the complex interactions between DBCI engagement, physical activity behavior, and individual context. With the pervasiveness of data loss in longitudinal studies, the results from this study also provide a use case for modeling idiographic data with differing levels of missing data via the *BayesLDM* toolbox. Lastly, the significant differences in DBN model results found at multiple levels of aggregation including across people, between groups, and between individual participants, highlights the benefits of an idiographic ‘first’ approach, particularly when it comes to identifying individual tailoring variables in pursuit of adaptive and effective prompt–and message-based interventions, while also exploring what works across similar individuals.

## Supplementary Material

Appendices_word.docx

## Data Availability

The study data are not publicly available.
