# Supplementary material for: A dynamic Bayesian network approach to modeling engagement and walking behavior: insights from a yearlong micro-randomized trial (Heartsteps II)
Source: Health Psychol Behav Med. 2025 Sep 18;13(1):2552479. doi: 10.1080/21642850.2025.2552479 (PMC12447456; doi:10.1080/21642850.2025.2552479)

**Appendices**

**Appendix A. Expanded *HeartSteps II* Study Description**

HeartSteps II Full Study Sample

The sample of *HeartSteps II* is comprised of overweight, but otherwise healthy adults (18-65) located Southern California (n=96). The mean age of the sample is 38 years, and the majority are female (n=73, 76%), married or in an intimate relationship (n=58, 60%), and working in a full-time job (n=60, 63%). The racial/ethnic composition of the *HeartSteps II* study is 19.8% Asian (n=19), 4% Black or African American (n=4), 2% American Indian / Alaska Native (n=2), 5% More than one race (n=5), 15.6% Other or Prefer not to respond (n=15), 2% Middle Eastern (n = 2), and 49% White (n=48). Lastly, 41.7% (n=40) reported being from a Hispanic/Latino ethnic background. In order to compare results from DBN models between Hispanic/Latinos and non-Hispanic/Latino Whites data was included from 10 participants (5 Hispanics and 5 non-Hispanic Whites). This collection of n-of-1 studies supports both idiographic (individual) and nomothetic (group level) comparisons by utilizing a “ground-up” approach to identify key variables for individuals and provide insights on “group-level” similarities and differences by race/ethnic.

Device & Measurement Considerations

Wearable activity trackers, specifically the Fitbit Versa, were used to passively detect and infer physical activity in the form of steps and minutes of activity. Through the *HeartSteps II* application, participants receive EMAs that query about the participant’s current contextual condition and psychological state. These EMAs act as “daily-check in” that measure a participant’s level of busyness, mood, motivation for and commitment to physical activity for that day. Participants also receive anti-sedentary messages when the wearable device detects inactivity from the user for an extended period of time (1 hour of consecutive inactive time). Lastly, participants received a weekly EMA prompt providing them with an opportunity to plan for exercise for the next week and asks the participant to reflect back to the previous week’s activity. Due to the micro-randomized trial (MRT) design of this study, these messages were determined for a particular user, rather than for the entire cohort. For example, a participant who is more active frequently will receive less anti sedentary messages than someone who spends more time inactive.

Study Measures

Psychosocial measures

- Perceived Busyness was measured by asking, “How busy is your day going to be today?”
- Perceived Restedness was measured by asking, “How well rested do you feel this morning?” Both questions were assessed on the morning EMA with responses ranging from not at all rested to very rested on a 5-point Likert scale.

Intervention components

- Messages Received was measured as the total number of daily prompts and messages received by the participant.

Passively detected measures

- Daily Fitbit Step Count Per Wear Time was calculated by taking the total number of daily steps detected by the Fitbit and dividing it by the amount of Fitbit wear time minutes per day.
- Temperature was measured passively by the *HeartSteps II* application based on the participants location and was aggregated to average temperature that day in degrees Fahrenheit.
- Application Page Views was detected passively by the *HeartSteps II* application and summed to the total daily application page views per day as used in our study. Engagement with DBCIs is dynamic, multidimensional, and multifaceted [56-60]. Behavioral engagement is typically collected passively and includes measures such as the number of app page views or the number of interactions with intervention components within the DBCI interface. Our study will focus primarily on app page views as an operationalization of app engagement.

Exogeneous measures

- Weekend was measured passively by deriving the participants study day of week (Saturday or Sunday) and then transformed into an indicator variable (1 = weekend, 0 = weekday).

All of the variables used in this study were standardized by taking the raw value, subtracting the mean and dividing by the standard deviation of the data series, also referred to as a z-score [61] There are several advantages in using standardized variables in our analyses including comparing effects from different types of variables.

Appendix B. Parameters Corresponding with Hypothesized Connections.

| *t_b* | Perceived Busyness [k-1] => Perceived Busyness [k] |
| --- | --- |
| *t_baa* | Perceived Busyness [k] => Application Page Views [k] |
| *t_baaa* | Perceived Busyness [k] => Fitbit Steps Per Wear Time Minute [k] |
| *t_fs* | Fitbit Steps Per Wear Time Minute [k-1] => Fitbit Steps Per Wear Time Minute [k] |
| *t_fsaa* | Application Page Views [k] => Fitbit Steps Per Wear Time Minute [k] |
| *t_m* | Messages Received [k] => Application Page Views [k] |
| *t_msfs* | Messages Received [k] => Fitbit Steps Per Wear Time Minute [k] |
| *t_pv* | Application Page Views [k-1] => Application Page Views [k] |
| *t_pvfs* | Application Page Views [k] => Fitbit Steps Per Wear Time Minute [k] |
| *t_r* | Perceived Restedness [k-1] => Perceived Restedness [k] |
| *t_raa* | Perceived Restedness [k] => Application Page Views [k] |
| *t_t* | Temperature [k] => Fitbit Steps Per Wear Time Minute [k] |
| *t_ww* | Weekend [k] => Application Page Views [k] |
| *t_www* | Weekend [k] => Fitbit Steps Per Wear Time Minute [k] |

**Appendix C. Full program example for idiographic DBN model**

program = """

ProgramName: Program1

Indices: k 0 347

Inputs: W, Temperature, MessagesReceived

t_b ~ N(0,1)

b_b ~ N(0,1)

s_b ~ Exp(0.1)

t_r ~ N(0,1)

b_r ~ N(0,1)

s_r ~ Exp(0.1)

t_pv ~ N(0,1)

t_baa ~ N(0,1)

t_raa ~ N(0,1)

t_ww ~ N(0,1)

t_m ~ N(0,1)

b_pv ~ N(0,1)

s_p ~ Exp(0.1)

t_fs ~ N(0,1)

t_fsaa ~ N(0,1)

t_baaa ~ N(0,1)

t_www ~ N(0,1)

t_t ~ N(0,1)

b_fs ~ N(0,1)

s_a ~ Exp(0.1)

t_msfs ~ N(0,1)

t_pvfs ~ N(0,1)

Busy[0] ~ N(0,1)

Rested[0] ~ N(0,1)

AppPageViews[0] ~ N(0,1)

stepwear[0] ~ N(0,1)

Busy[k] ~ N(t_b * Busy[k-1] + b_b, s_b)

Rested[k] ~ N(t_r * Rested[k-1] + b_r, s_r)

AppPageViews[k] ~ N(t_pv * AppPageViews[k-1] + t_baa * Busy[k] + t_raa * Rested[k] + t_ww * W[k] + t_m * MessagesReceived[k] + b_pv, s_p)

stepwear[k] ~ N(t_fs * stepwear[k-1] + t_fsaa * AppPageViews[k] + t_pvfs * AppPageViews[k-1] + t_baaa * Busy[k] + t_www * W[k] + t_msfs * MessagesReceived[k] + t_t * Temperature[k] + b_fs, s_a)""

Appendix D. Full MCMC Parameter Estimates Across Participants


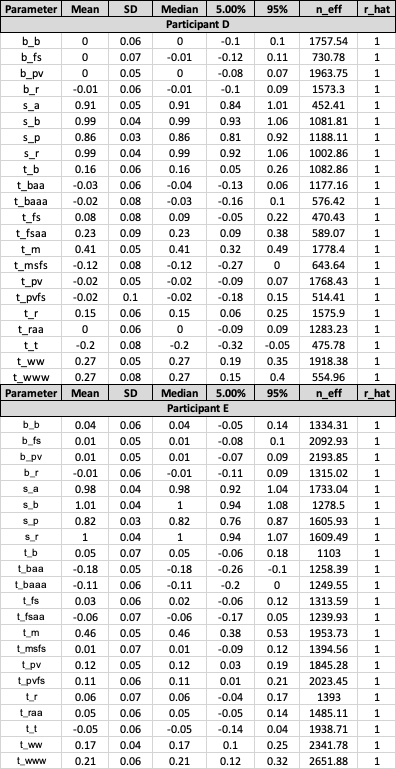

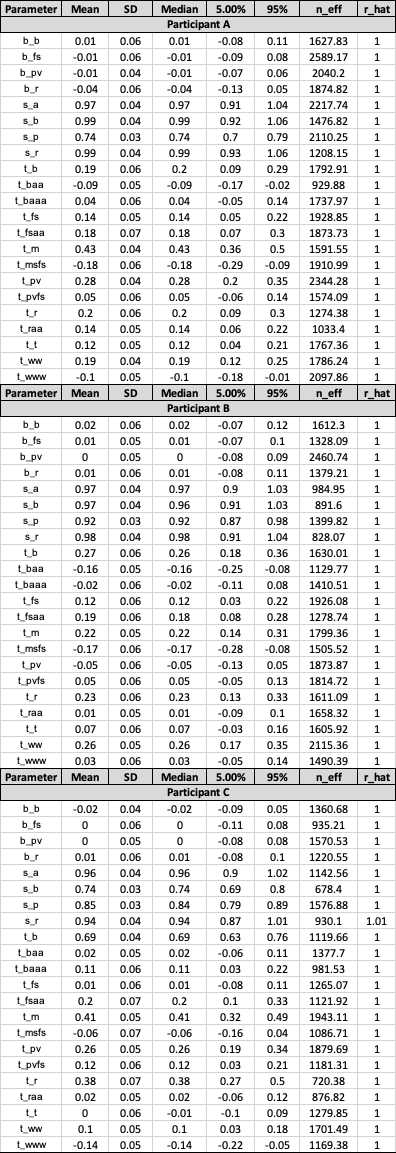


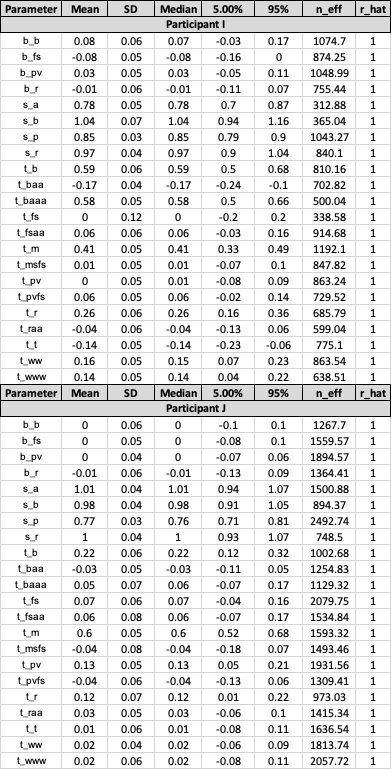


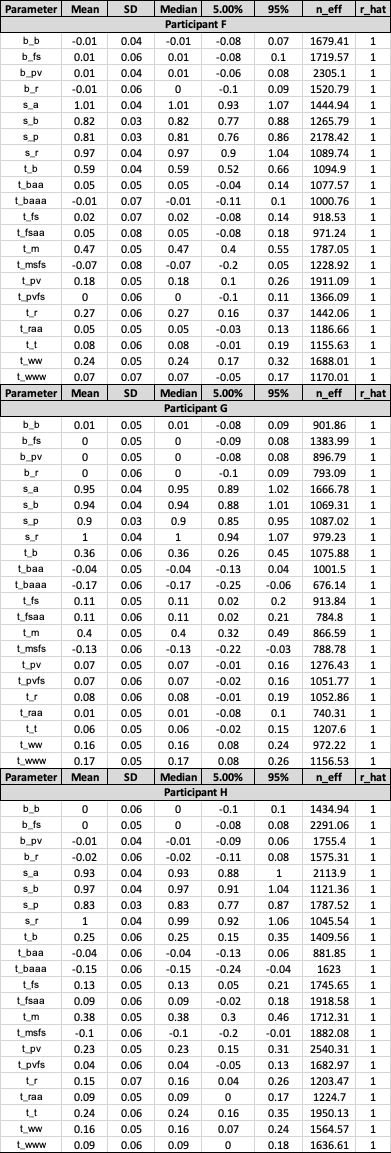


Appendix E.

**Figure Appendix E. Caption:** Posterior Distributions of the Impact of Messages & Prompts Received by the Participant [k] on App Page Views [k] at the same timepoint.

**Figure Appendix E. Alt Text:** A grid of posterior distributions comparing the effects of messaging on app page views across Hispanic/Latino and non-Hispanic/Latino White participants. Each plot shows the posterior mean and distribution.

**Figure Appendix E. Alt Text (Long Description):** A series of histograms displaying posterior distributions for individual participants, grouped by ethnicity. The top row contains five plots for Hispanic/Latino participants (letters A to E), while the bottom row shows five plots for non-Hispanic/Latino White participants (letters F to J). Each plot features a blue histogram representing the posterior distribution of the effect of messages received on app page views, with a red vertical line indicating the posterior mean. The data suggests varying effects among participants across both groups.


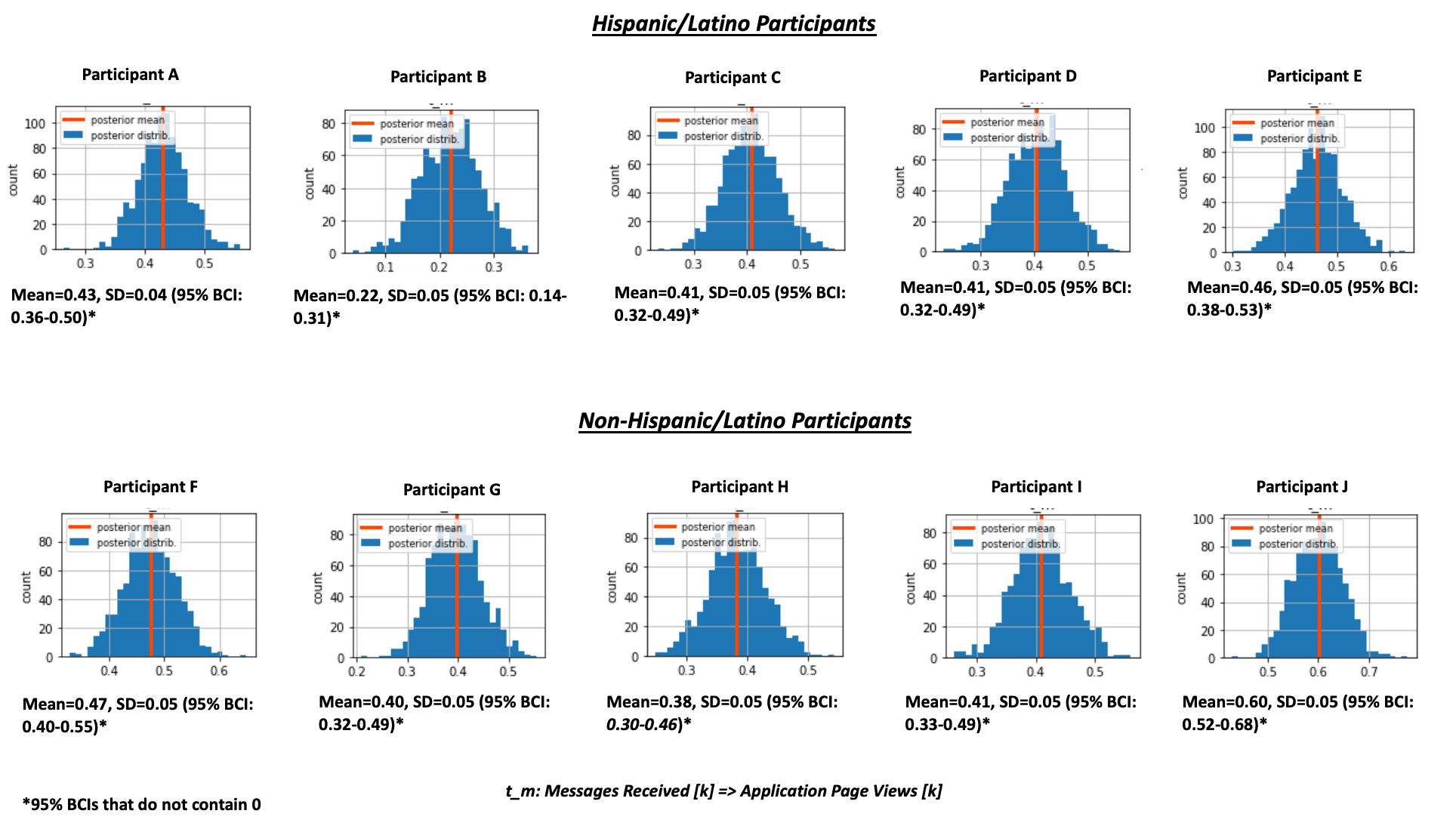


Appendix F.

**Appendix F. Caption:** Posterior Distributions of the Impact of App Page Views [k] on Fitbit Steps Per Wear Time [k] at the same timepoint

**Appendix F. Alt Text**: A grid of posterior distributions showing the relationship between app page views and daily Fitbit steps per wear time minute for Hispanic/Latino and non-Hispanic/Latino participants. Each plot displays the posterior mean and distribution.

**Appendix F. Alt Text (Long Description):** A series of histograms showing posterior distributions for participants, grouped by ethnicity. The top row includes five plots for Hispanic/Latino participants (letters A to E), and the bottom row includes five plots for non-Hispanic/Latino participants (letters F to J). Each plot features a blue histogram representing the posterior distribution of the effect of app page views on daily Fitbit steps per wear time minute, with a red vertical line indicating the posterior mean. The distributions highlight varying idiographic effects across both groups.


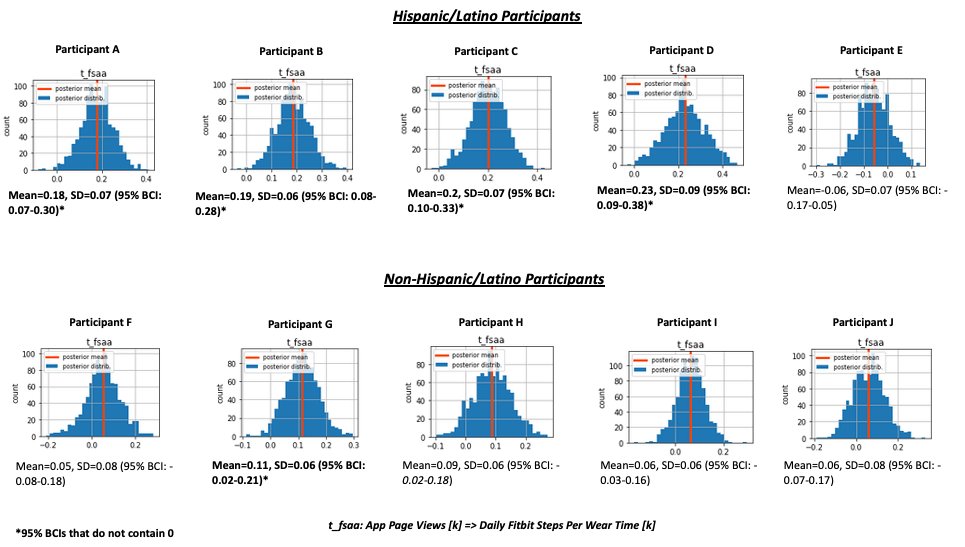

Supplement: Appendices_word.docx [file RHPB_A_2552479_SM6447.docx]
